# Urokinase-type plasminogen activator receptor inhibits apoptosis in triple-negative breast cancer through miR-17/20a suppression of death receptors 4 and 5

**DOI:** 10.18632/oncotarget.20435

**Published:** 2017-08-24

**Authors:** Xin Li, Bo Wu, Lizhao Chen, Ying Ju, Changfei Li, Songdong Meng

**Affiliations:** ^1^ CAS Key Laboratory of Pathogenic Microbiology and Immunology, Institute of Microbiology, Chinese Academy of Sciences (CAS), Beijing, China; ^2^ College of Life Sciences, University of Chinese Academy of Sciences, Beijing, China

**Keywords:** uPAR, miR-17-5p, miR-20a, DR5, apoptosis

## Abstract

Dissection and understanding of the molecular pathways driving triple-negative breast cancer (TNBC) are urgently needed to develop efficient tailored therapies. Aside from cell invasion and metastasis, the urokinase-type plasminogen activator receptor (uPAR) has been linked to apoptosis resistance in breast tumors. We explored the mechanism of uPAR-disrupted apoptosis in breast cancer. We found that depletion of uPAR by RNAi increases death receptor 4 (DR4) and death receptor 5 (DR5) expression and triggers TRAIL-induced apoptosis in TNBC cells. The microRNAs miR-17-5p and miR-20a inhibit cell apoptosis via suppression of DR4/DR5. We provide evidence that uPAR enhances miR-17-5p/20a expression through upregulation of c-myc. Blocking miR-17-5p/20a with antagomiRNA suppressed the growth of uPAR-overexpressing breast tumor xenografts in mice. These results indicate that uPAR suppresses cell apoptosis by inhibiting the c-myc-miR-17/5p/20a-DR4/DR5 pathway. Therapy directed at uPAR-induced miR-17/20a is a potential option for breast cancer and TNBC.

## INTRODUCTION

Urokinase plasminogen activator receptor (uPAR) (also designated CD87) is a highly glycosylated membrane-anchored protein. Along with its ligand urokinase-type plasminogen activator (uPA), uPAR is a signaling receptor that interacts with proteins such as integrins, vitronectin, LRP-related receptor, and others [3]. An important function of the uPA-uPAR system in cancer progression is its activity in the proteolysis of the extracellular matrix (ECM). Pro-uPA, a zymogen of uPA, binds to uPAR and is converted to uPA, which cleaves plasminogen to active plasmin. The components of the surrounding ECM are degraded by plasmin, and many promatrix metalloproteinases (MMPs) are activated and released, thus promoting tumor cell invasion and metastasis [1, 2]. In addition, as a glycosylphosphatidylinositol-anchored (GPI-anchored) cell surface protein, uPAR relays its downstream signals via its co-receptors, including integrins and growth factor receptors (GFRs). These interactions activate the FAK, Src, ERK, and PI3K/AKT signaling pathways, which might induce an epithelial to mesenchymal transition (EMT), cell proliferation, and migration [3-7].

uPAR is overexpressed in a variety of cancers, including breast cancer, and its presence is associated with poor prognosis [8-11]. Because uPAR promotes tumor progression and invasion, various strategies for blocking uPA-uPAR interaction or uPAR-mediated downstream signaling are being developed to inhibit tumor growth and metastasis [12-15].

uPAR has been demonstrated to inhibit apoptosis in glioma and colon cancer via the Bcl-2 and JNK-p53 signaling pathways [16, 17]. In a previous study, we found that downregulation of uPAR induces apoptosis in breast cancer cells, but the mechanism behind this phenomenon deserves further investigation [18]. Because pro-apoptotic and anti-apoptotic signals are factors in the induction of breast tumorigenesis and acquired resistance to treatments [19], the aim of the present study was to explore the potential mechanisms of downregulation of uPAR-induced apoptosis in breast cancer. Our study reveals a new underlying miR-17-5p/20a- mediated pathway by which uPAR induces cell apoptosis in breast cancer. Blocking that pathway is a potential option for breast cancer therapy.

## RESULTS

### uPAR depletion induces apoptosis and increases death receptor 4 and death receptor 5 levels

**Figure 1:**
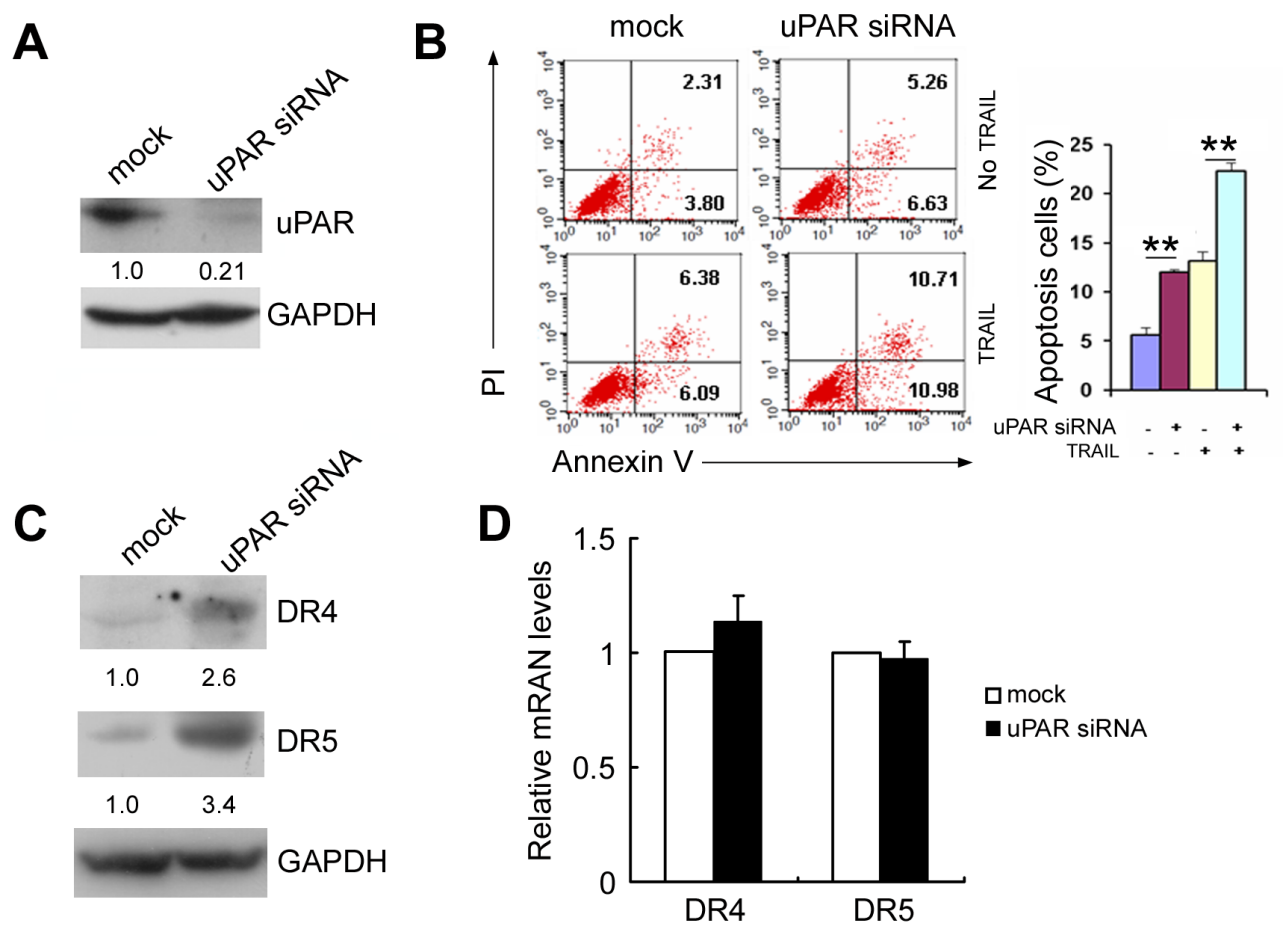
uPAR siRNA induces apoptosis and increases DR4 and DR5 expression MDA-MB-231 cells were transfected with uPAR siRNA or a control siRNA as a mock. **(A)** At 48 hours after transfection, uPAR protein levels were detected by Western blotting. **(B)** At 48 hours after transfection, cells were treated with or without 50 ng/mL TRAIL for an additional 8 hours, and then stained with Annexin V/FITC and PI for cellular apoptosis detection. The percentage of apoptotic cells (Annexin V single positive and Annexin V/PI double positive) was assessed. **(C)** At 48 hours after transfection, DR4 and DR5 protein levels were detected by Western blotting. **(D)** At 24 hours after transfection, DR4 and DR5 mRNA levels were detected by real-time PCR. Data are presented as means ± SD from three independent experiments.

Similar to the results of a previous study [18], depletion of uPAR by siRNA induced apoptosis in triple negative MDA MB 231 cells that express relatively high levels of uPAR (Figure 1A and 1B). In addition, treatment with uPAR siRNAi increased tumor necrosis factor-related apoptosis-induced ligand-induced (TRAIL-induced) cell apoptosis by approximately1.6-fold (Figure 1B). Because TRAIL binds to the pro-apoptotic death receptor 4 (DR4) and death receptor 5 (DR5) and triggers the cell apoptosis pathway [21], we determined whether uPAR depletion influences DR4 and DR5. As seen in Figure 1C, treatment with uPAR siRNA caused approximately 2.6-fold and 3.4-fold increases, respectively, in DR4 and DR5 levels in MDA MB 231 cells. However, uPAR depletion did not increase DR4 and DR5 mRNA levels (Figure 1D).

### MiR-17-5p/20a inhibits TRAIL-induced apoptosis by suppressing DR4 and DR5 in breast cancer cells

**Figure 2:**
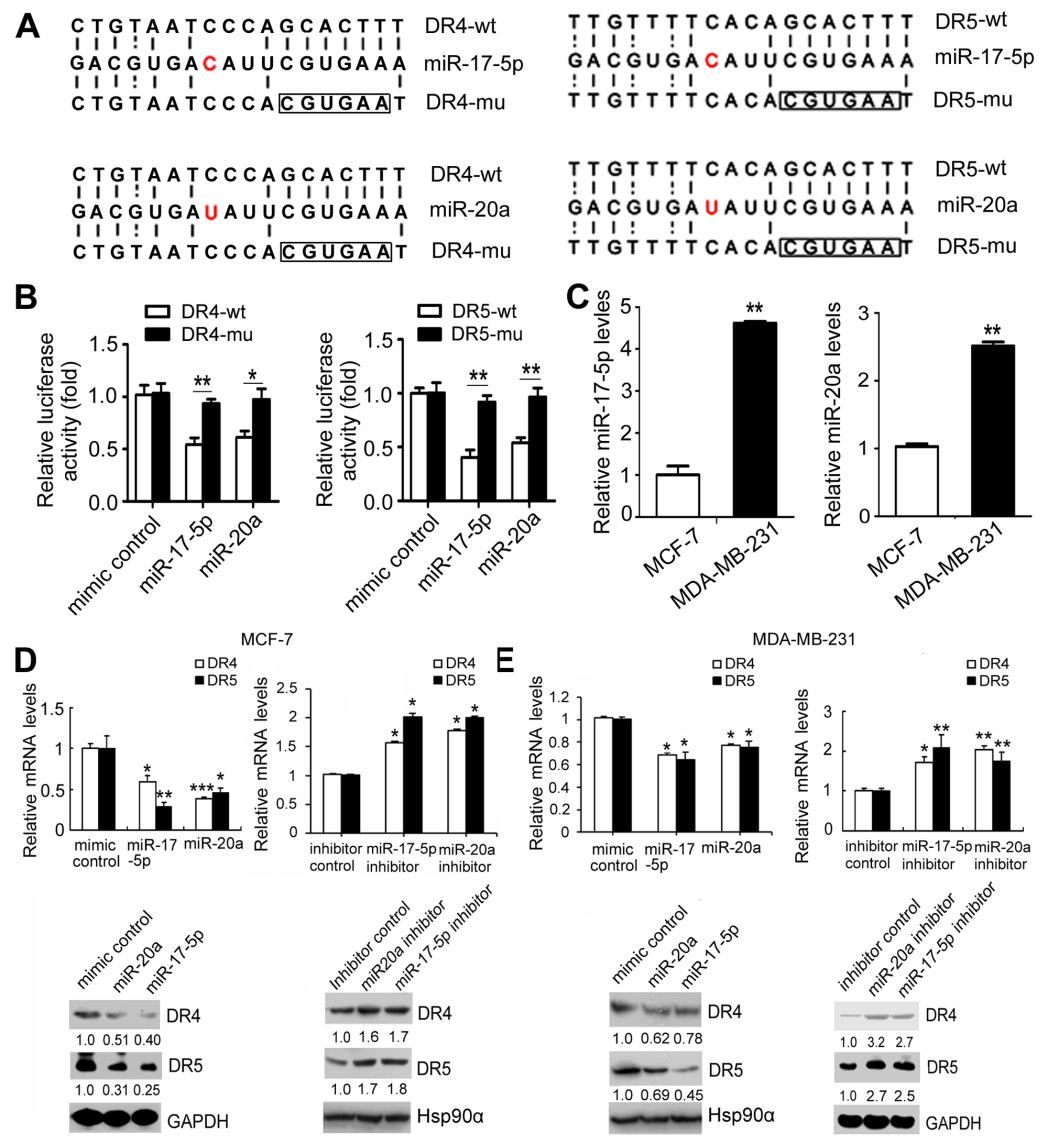
DR4 and DR5 are suppressed by of miR-17-5p/20a **(A)** Perfect matches are indicated by dark vertical lines. Mutations in the seed region of the miR-17-5p (up) or miR-20a (lower) binding sites in the DR4 (nucleotides 1630-1647) or DR5 UTR (nucleotides 1892-1909) are marked in the box. **(B)** Luciferase activity measured in 293T cells co-transfected with miR-17-5p or miR-20a mimic or a randomized oligonucleotide as a control and a firefly luciferase reporter containing either the wild-type (DR4-wt or DR5-wt) or mutant miR-17-5p and miR-20a binding sites (DR4-mu or DR5-mu). **(C)** miR-17-5p (left) and miR-20a (right) levels in MCF-7 and MDA MB 231 cells were detected by real-time PCR. **(D** and **E)** MCF-7 cells (D) or MDA MB 231 cells (E) were transfected with miR-17-5p or miR-20a mimic, miR-17-5p or miR-20a inhibitor or a randomized oligonucleotide as a control. DR4 and DR5 mRNA (up panels) and protein levels (low panels) were determined by real-time PCR and Western blotting at 24 hours and 48 hours after transfection, respectively. Data are presented as means ± SD from three independent experiments. ^*^P < 0.05, ^**^P < 0.01 and ^***^P < 0.001 (compared with control in Figure 2D and Figure 2E).

Because microRNAs (miRNAs) post-transcriptionally inhibit approximately half of the human transcriptome via degradation and translational repression of target mRNAs [22], we speculated that uPAR might inhibit DR4 and DR5 expression via miRNAs. We screened miRNAs that have binding sites in the DR4 or DR5 3′-UTR by use of TargetScan (http://www.targetscan.org/vert_71/). The miR-17-5p and miR-20a miRNAs were selected from the top 40 miRNAs according to context score percentiles, because they belong to the miR-17-92 cluster that is involved in cell proliferation and apoptosis [23, 24, 25] and they bind to both DR4 and DR5. As seen in Figure 2A, perfect matches of the seed sequence are shown by vertical lines between the DR4 3′-UTR (nucleotides 1630–1647) or DR5 3′-UTR (nucleotides 1892–1909) and miR-17-5p or miR-20a. Mutations were made in the seed region of the miR-17-5p/20a binding site as a control. The miR-17-5p/20a miRNAs reduced the activity of a firefly luciferase reporter by binding to the wild-type (DR4-wt or DR5-wt) but not the mutant (DR4-mut or DR5-mut) DR4 or DR5 3′UTR (Figure 2B), confirming that miR-17-5p/20a interacts with this binding site. We further examined the influence of miR-17-5p/20a on DR4 or DR5 expression in breast cancer cells. The expression levels of miR-17-5p/20a are relatively high in MDA-MB-231 cells and low in MCF-7 cells (Figure 2C). Treatment with miR-17-5p/20a mimics caused a decrease in DR4 or DR5 mRNA, whereas miR-17-5p/20a inhibitors caused an increase in DR4 or DR5 mRNA (Figure 2D and 2E) and protein (Figure 2D and 2E) expression levels in these two cells, indicating that both DR4 and DR5 are inhibited by miR-17-5p/20a.

**Figure 3:**
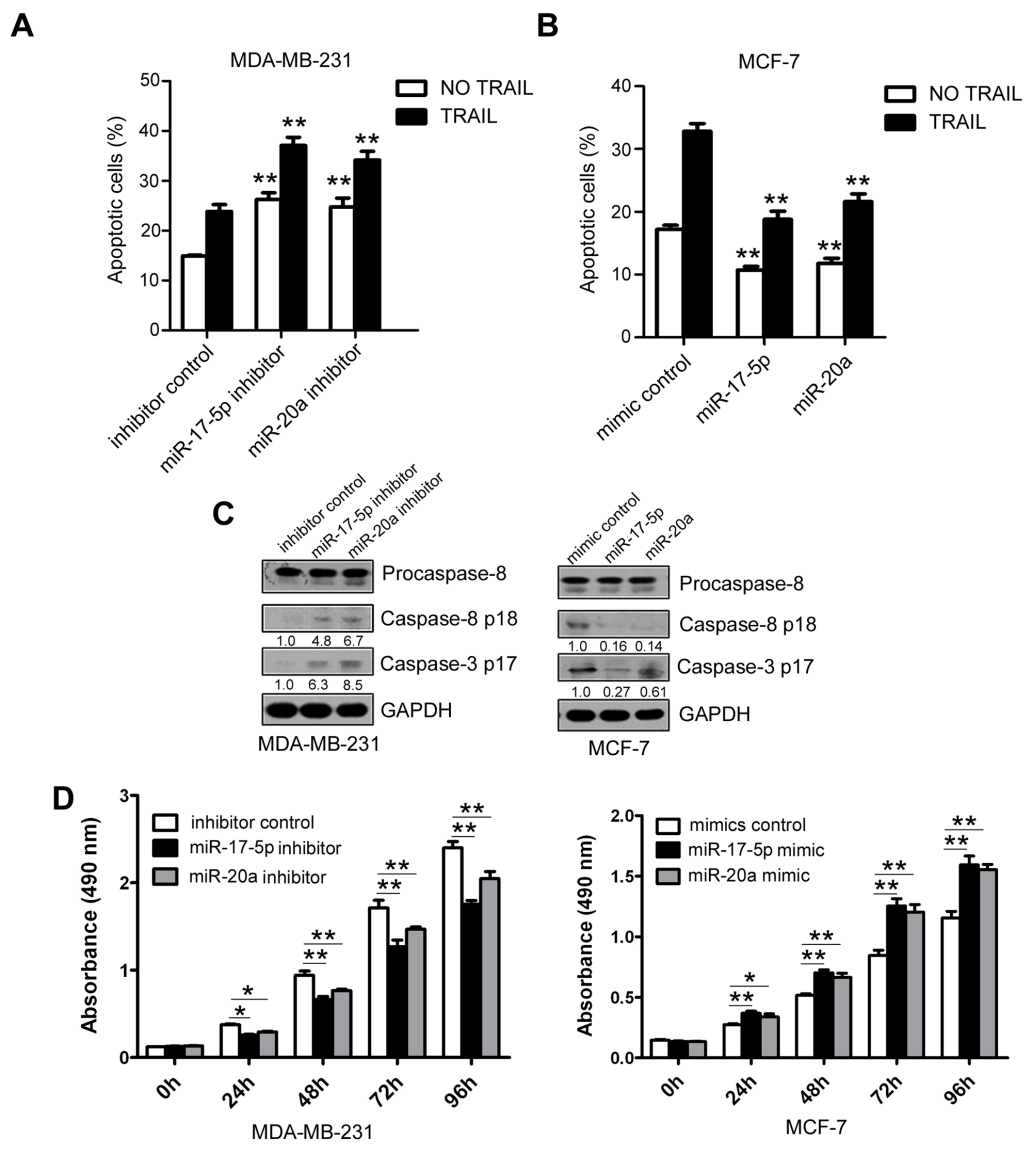
Inhibition of TRAIL-induced apoptosis by miR-17-5p/miR-20a in breast cancer cells **(A–D)** MDA MB 231 cells were transfected with miR-17-5p or miR-20a inhibitor or a randomized oligonucleotide as an inhibitor control, and MCF-7 cells were transfected with miR-17-5p or miR-20a mimic or a randomized oligonucleotide as a mimic control. At 48 hours after transfection, cells were treated with or without 50 ng/mL TRAIL for an additional 48 hours, and apoptotic MDA MB 231 cells (A) and MCF-7 cells (B) were detected by FACS as in Figure 1A. Expression of procaspase-8, caspase 8 p18, and caspase 3 p17 was analyzed in MDA MB 231 (left) and MCF-7 cells (right) by Western blotting (C). The cell growth of MDA MB 231 cells (left) and MCF-7 cells (right) was examined by CCK-8 assays at 24, 48, 72, and 96 hours after transfection (D). Data are presented as means ± SD from three independent experiments. ^*^P < 0.05, ^**^P < 0.01 and ^***^P<0.001 (compared with control in Figure 3A and Figure 3B).

Because DR4 and DR5 trigger the extrinsic apoptosis pathway on binding to its ligand TRAIL by cleavage and activation of the effectors caspase-8 and caspase-3 [26], we investigated whether miR-17-5p/20a increase cell apoptosis in breast cancer. Transfection of MDA MB 231 cells, which express relatively high levels of miR-17-5p and miR-20a, with miR-17-5p or miR-20a inhibitors significantly induced apoptosis (without TRAIL) and increased TRAIL-induced cell apoptosis compared with controls (both *P* < 0.01) (Figure 3A and Supplementary Figure 2A). Conversely, transfection of their mimics in MCF-7 cells, which express relatively low levels of miR-17-5p and miR-20a, suppressed apoptosis (without TRAIL) and TRAIL-induced cell apoptosis (all *P* < 0.01) (Figure 3B and Supplementary Figure 2B). Treatment with miR-17-5p or miR-20a inhibitor abruptly increased the protein levels of activated caspase 8 and caspase 3, whereas treatment with miR-17-5p or miR-20a mimics decreased the levels of caspase 8 and caspase 3 (Figure 3C). CCK-8 assays revealed that treatment with miR-17-5p or miR-20a inhibitor resulted in decreased cell growth, whereas an miR-17-5p or miR-20a mimic caused an increase in cell growth (all *P* < 0.05) (Figure 3D).

**Figure 4:**
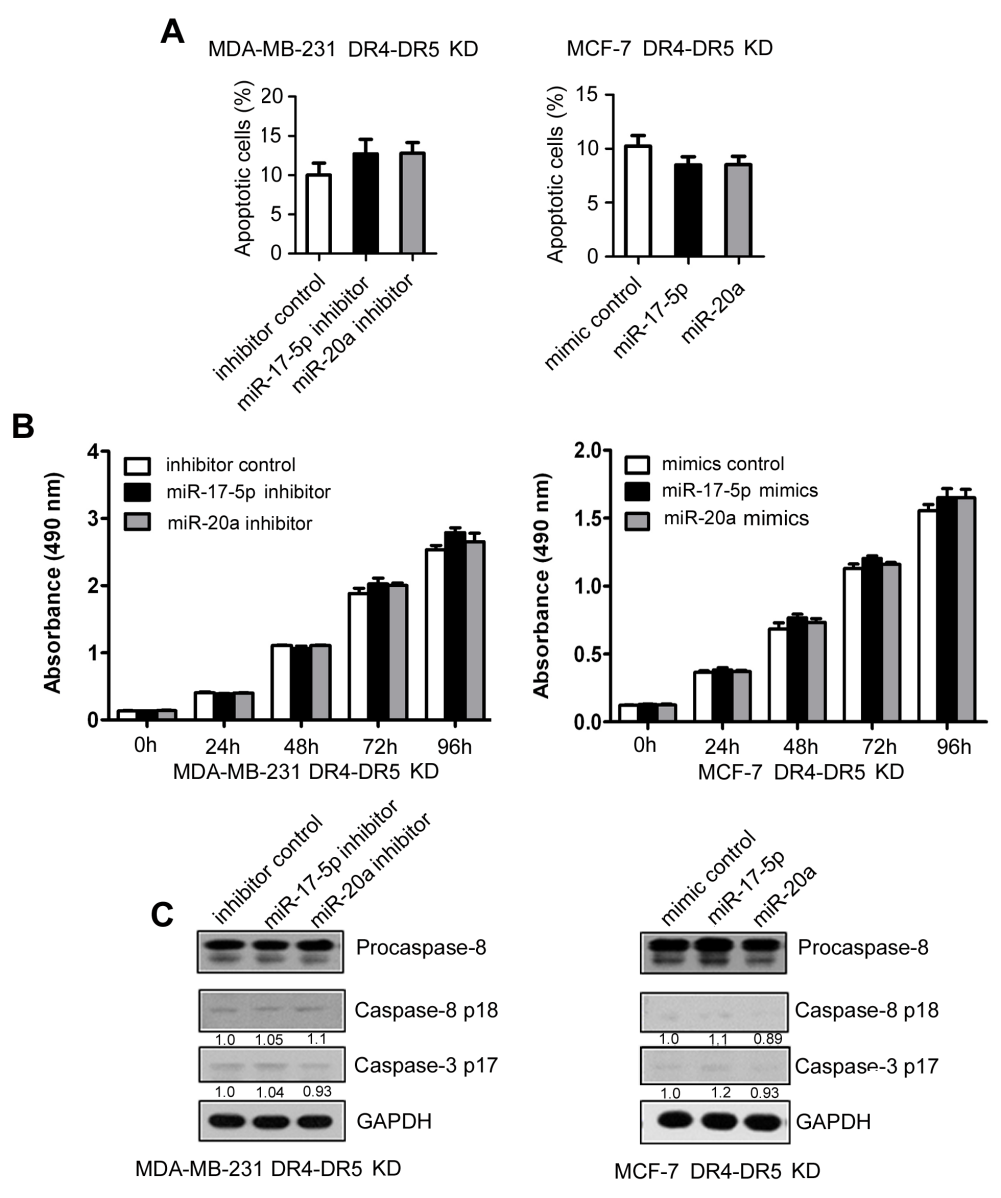
Inhibition of TRAIL-induced apoptosis by miR-17-5p/20a through DR4 and DR5 in breast cancer cells **(A–C)** MDA MB 231 cells were co-transfected with DR4/DR5 siRNA and miR-17-5p or miR-20a inhibitor or a randomized oligonucleotide as an inhibitor control, and MCF-7 cells were co-transfected with DR4/DR5 siRNA and miR-17-5p or miR-20a mimic or a randomized oligonucleotide as a mimic control. At 48 hours after transfection, cells were treated with 50 ng/mL TRAIL for an additional 8 hours, and then apoptotic MDA MB 231 cells (left) and MCF-7 cells (right) were detected by FACS as in Figure 1A (A). The cell growth of MDA MB 231 cells (left) and MCF-7 cells (right) was examined by CCK-8 assays at 24, 48, 72, and 96 hours after transfection (B). Expression of procaspase-8, caspase 8 p18, and caspase 3 p17 was analyzed in MDA MB 231 (left) and MCF-7 cells (right) by Western blotting (C). Data are presented as means ± SD from three independent experiments. ^*^P < 0.05 and ^**^P < 0.01.

To further determine whether miR-17-5p/20a inhibits apoptosis and cell growth by suppressing DR4 and DR5, miR-17-5p-expressing or miR-20a-expressing cells were co-transfected with DR4 and DR5 siRNA. The effects of miR-17-5p and miR-20a inhibitors or mimics on cell apoptosis (Figure 4A and Supplementary Figure 1) and cell growth (Figure 4B) were largely attenuated by DR4 and DR5 depletion. Similarly, miR-17-5p and miR-20a inhibitors or mimics could not cause a change in activated caspase 8 and caspase 3 levels in DR4 and DR5 siRNA-treated cells (Figure 4C). These data suggest that the inhibition of cell apoptosis by miR-17-5p/20a is largely dependent on suppression of DR4 and DR5.

### uPAR upregulates c-myc-induced miR-17-5p/20a expression

**Figure 5:**
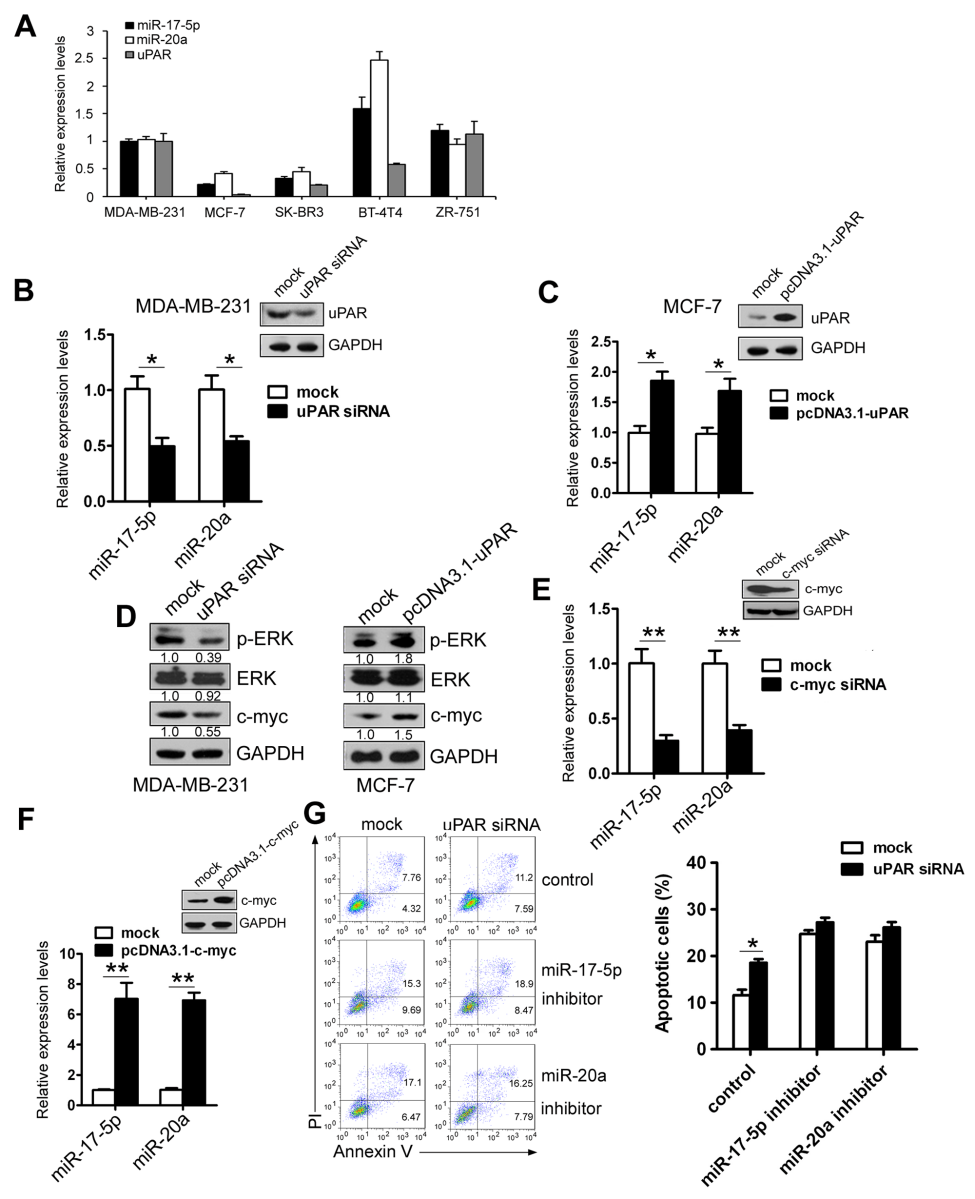
uPAR upregulates miR-17-5p/20a expression via c-myc **(A)** uPAR, miR-20a, and miR-17-5p levels in MDA MB 231, MCF-7, SK-BR3, BT-4T4, and ZR-751 cells were detected by real-time PCR. **(B** and **C)** MDA MB 231 (B) or MCF-7 cells (C) were transfected with uPAR siRNA (a control siRNA as a mock) or pcDNA3.1-uPAR (pcDNA3.1 as a mock). At 48 hours after transfection, miR-17-5p or miR-20a levels were detected by real-time PCR. uPAR protein levels in uPAR siRNA- or pcDNA3.1-uPAR-treated MDA MB 231 (B) or MCF-7 cells (C) were analyzed by Western blotting. **(D)** MDA MB 231 (left) or MCF-7 cells (right) were transfected with uPAR siRNA or pcDNA3.1-uPAR. Expression of P-ERK, ERK, and c-myc was detected by Western blotting at 48 hours after transfection. **(E** and **F)** MDA MB 231 (E) or MCF-7 cells (F) were transfected with c-myc siRNA (a control siRNA as a mock) or pcDNA3.1-c-myc (pcDNA3.1 as a mock). miR-17-5p or miR-20a levels were detected by real-time PCR, and c-myc protein levels were determined by Western blotting at 48 hours after transfection. **(G)** MDA MB 231 cells were co-transfected with uPAR siRNA or a control siRNA as a mock and miR-17-5p or miR-20a inhibitor (a randomized oligonucleotide as a control). Cellular apoptosis was assayed by FACS at 48 hours after transfection. At 48 hours after transfection, cells were treated with 50 ng/mL TRAIL for an additional 8 hours, and then stained with Annexin V/FITC and PI. Cellular apoptosis was determined by FACS as in Figure 1A. Data are presented as means ± SD from three independent experiments. ^*^P < 0.05 and ^**^P < 0.01.

Five breast cancer cell lines were selected to detect uPAR mRNA and miR-17-5p/20a levels by real-time PCR. As shown in Figure 5A, a similar expression of uPAR and miR-17-5p/20a occurred in these cell lines except in the BT-474 cell lines, in which higher levels of miR-17-5p/20a occurred with higher uPAR levels and vice versa. Transfection with uPAR siRNA in MDA MB 231 cells with uPAR overexpression led to a decrease in miR-17-5p/20a levels (Figure 5B). Conversely, overexpression of uPAR in the poorly uPAR-expressing MCF-7 cells resulted in an increase in miR-17-5p/20a levels (Figure 5C).

We examined the mechanism of uPAR-induced miR-17-5p/20a upregulation. As seen in Figure 5D, phosphorylated ERK and c-myc levels were decreased in MDA MB 231 cells transfected with uPAR siRNA. However, uPAR overexpression in MCF-7 cells caused an increase in phosphorylated ERK and c-myc protein levels. Because a previous study demonstrates that c-myc binds to the miR-17-cluster locus and enhances miR-17 and miR-20a expression [27], we speculated that uPAR might induce miR-17-5p/20a expression via c-myc. As seen in Figure 5E, c-myc depletion by siRNA led to a decrease in the levels of miR-17-5p by 70% and miR-20a by 60% compared with mock-treated cells. Overexpression of c-myc in MCF-7 cells resulted in an increase in miR-17-5p by 7.0-fold and miR-20a levels by 6.9-fold (Figure 5F). No differences in cell apoptosis were observed between uPAR siRNA-treated and control siRNA-treated cells under miR-17-5p/20a depletion by inhibitors (Figure 5G). Collectively, these data indicate that uPAR enhances miR-17-5p/20a expression by c-myc and thus inhibits cell apoptosis.

### Inhibition of miR-17-5p and miR-20a levels by antagomir-17-5p and antagomir-20a delivery induces apoptosis and suppresses breast tumor growth in nude mice

**Figure 6:**
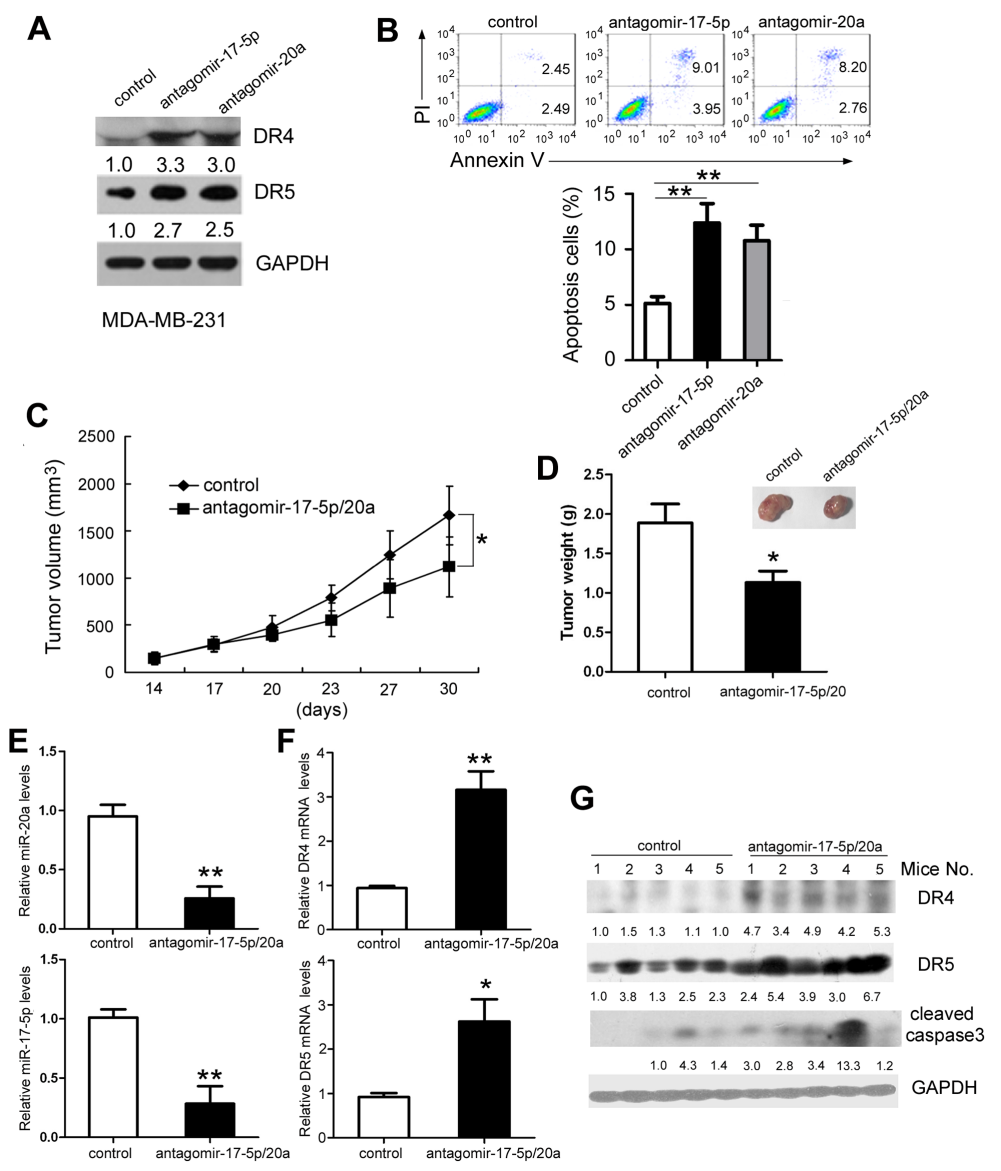
Inhibition of miR-17-5p and miR-20a expression suppresses tumor growth in nude mice **(A** and **B)** MDA MB 231 cells were treated with cholesterol-conjugated miR-17-5p and miR-20a inhibitors or a cholesterylated randomized oligonucleotide as a control. At 48 hours after treatment, DR4 and DR5 protein levels (A) and cellular apoptosis were analyzed by FACS as in Figure 1A (B). **(C–G)** Female BABL/c-nu mice were subcutaneously inoculated with 7 × 10^6^ MDA MB 231 cells. When the tumor volume reached 150 mm^3^, the mice were randomly divided into two groups (5 mice/group). Cholesterol-conjugated miR-17-5p and miR-20a inhibitor (10 nmol/mouse) or cholesterylated randomized oligonucleotide were intratumor-delivered five times every 3 days. (C) The tumor diameters were measured every 3 days. (D) Three days after the last intratumor injection, all mice were sacrificed, and the tumors were excised. The tumor weight was measured. Tumors were photographed and shown. (E–G) miR-20a (E, left), miR-17-5p (E, right), and DR4 and DR5 mRNA (F) in the tumors of the two groups were detected by real-time PCR. Activated caspase 3 and DR4/DR5 protein levels were determined by Western blotting (G). Data are presented as means ± SD from three independent experiments. ^*^P < 0.05 and ^**^P < 0.01.

We assessed whether inhibiting uPAR-induced miR-17-5p/20a could suppress growth of triple negative MDA-MB-231 tumor xenografts that highly express uPAR. Antagomir-17-5p and antagomir-20a are synthesized cholesterylated stable miR-17-5p and miR-20a inhibitors that have two oxygen methylation modifications and a sulfur-modified phosphate. Antagomir-17-5p and antagomir-20a increased DR4 and DR5 protein levels (Figure 6A) and induced apoptosis in MDA MB 231 cells (Figure 6B). Because similar apoptosis-inducing activity was observed *in vitro* between antagomir-17-5p and antagomir-20a, MDA MB 231-xenografted nude mice were injected with these two antagomirs simultaneously to obtain better antitumor activity. As shown in Figure 6C, tumor growth was suppressed in the antagomir-17-5p/20a-treated group compared with controls (*P* < 0.05). Antagomir-17-5p/20a treatment caused a decrease in tumor weight by 40% compared with tumors in control mice (*P* < 0.05) (Figure 6D). Representative photographs of the tumors in the two groups are shown in Figure 6D. Depletion of miR-20a and miR-17-5p (Figure 6E) and upregulation of DR4/DR5 mRNA and protein levels (Figure 6F and 6G) in tumors were verified by real-time PCR and Western blotting, respectively. Additionally, increased cleaved caspase 3 was also observed in antagomir-17-5p/20a-treated tumors (Figure 6G)

## DISCUSSION

uPAR is both an anchor for uPA and involved in intracellular signal transduction events. Our previous study demonstrates that uPAR downregulation induces apoptosis in breast cancer. In the current study, we explored the molecular mechanisms of uPAR inhibition of apoptosis in breast cancer. We found that uPAR induces miR-17-5p and miR-20a expression by upregulating the transcription factor c-myc, whereas miR-17-5p/20a inhibit breast cancer apoptosis by suppressing DR4 and DR5. Thus, uPAR acted through the miR-17/5p/20a- DR4/DR5 pathway to block cell apoptosis. miR-17-5p/20a antagomirs inhibited the growth of triple-negative breast tumor xenografts in nude mice. These *in vitro* and *in vivo* experiments suggested that uPAR contributes to resistance to tumor apoptosis and that directing therapy at uPAR-induced miR-17-5p/20a is a potential therapeutic option in breast cancer.

Because TNBC lacks overexpression of estrogen receptor (ER), progesterone receptor (PR), and human epidermal growth factor receptor (HER2) and no effective drugs are currently available, the biology of this complex cancer must be discerned to develop specific therapeutic strategies to improve patient survival [28, 29]. In breast cancer, elevated uPAR expression is an independent prognostic marker of shortened relapse-free survival, metastases-free survival, and overall survival [30-32], although specific therapeutic agents have not been developed for these uPAR-overexpressing patients. uPAR overexpression is observed in a substantial proportion of TNBCs [33]. In this study, we found that uPAR knockdown increased the pro-apoptotic DR4 and DR5 protein levels in the MDA MB 231 TNBC cell line and confirmed that DR4 and DR5 are suppressed by miR-17-5p and miR-20a, which is similar to the results of Krishnamoorty et al. [34] showing that uPAR-depleted glioma cells have higher levels of DR4 and DR5. We focused on miR-17-5p and miR-20a in this study because they belong to the same miRNA cluster (miR-17-92 cluster), which has strong oncogenic potential [23, 24, 25]. The miR-17-92 cluster includes six miRNAs (miR-17, miR-18a, miR-19a, miR-20a, miR-19b-1, and miR-92-1) that are located within an 800-bp region of human chromosome 13. The miR-17 and miR-20a miRNAs are usually expressed as miR-17/20a because they share an identical sequence in both humans and mice [35, 36]. Our data demonstrated that uPAR upregulated miR-17 and miR-20a expression via c-myc, and uPAR reduced cell apoptosis by increasing miR-17-5p/ 20a expression, which caused inhibition of TRAIL-induced apoptosis.

This report is the first showing that uPAR induces miR-17 and miR-20a expression. Blocking uPAR-induced miR-17-5p/20a by antagomir treatment significantly attenuated TNBC tumor growth in mice. These results suggest that disrupting uPAR-induced miR-17/20a is a potential therapeutic option for TNBC cancer. Besides inhibition of TRAIL-induced apoptosis, miR-17/20a enhances cell proliferation (see Figure 3D), which might be the result of suppression of other factors. Therefore, miR-17/20a antagomir-mediated tumor inhibition in mice might not be totally attributable to antagomir-induced cell apoptosis.

We determined that DR4 and DR5 are suppressed by miR-17-5p/20a, which blocks cell apoptosis in breast cancer. The miR-17-92 cluster is overexpressed in breast, liver, colon, pancreas, and other types of cancer [23, 25, 37-39]. A previous report demonstrates that miR-17-92 contributes to B-cell lymphomagenesis, indicating oncogenic activity [23]. Subsequent studies show that miR-17-92 is a bona fide oncogene and is causatively oncogenic [24, 40, 41]. The identified genes that are suppressed by miR-17-92 include the E2F family, c-myc, cyclin-dependent kinase inhibitor CDKN1A (p21), and the pro-apoptotic gene BCL2L11/BIM. These genes are critical factors in the cell cycle, apoptosis, and tumor angiogenesis, and they are involved in the processes of miR-17-92, contributing to tumorigenesis [27, 42, 43, 44-48]. Another important gene that is suppressed by miR-17-92 is PTEN [37, 49], and a recent study confirms that TGFBR2 is suppressed by miR-17-5p/20a [50]. These genes have tumor-suppressive functions, validating that directing therapy at the miR-17-92 cluster miRNAs is a therapeutic option for various types of cancer. Given the multiple functions of the miR-17-92 cluster in the cell cycle, apoptosis, and tumorigenesis, the finding that uPAR induces miR-17-5p/20a expression might broaden our knowledge of the function of uPAR in cancer development, progression, and metastases.

Downregulation of uPAR leads to the sensitization of colon cancer cells to TRAIL-induced apoptosis via active p53 and mitochondrial apoptotic pathways [17]. Additionally, uPAR downregulation might induce apoptosis in gliomas through calcineurin redistribution, its reduced association with Bcl-2, and the dephosphorylation of the Bcl2-associated death promoter (BAD) [16]. However, in our model system, downregulation of uPAR increased TRAIL-induced apoptosis through induction of the expression of miR-17-5p and miR-20a and inhibition of DR4 and DR5. The uPAR-inhibited cell apoptosis was largely blocked by depletion of miR-17-5p/20a or by DR4 and DR5 in breast cancer cells (see Figure 4 and Figure 5). These data also highlight that inhibition of cell apoptosis by uPAR can be tumor-type specific, which might be the result of different expression profiles and interactions of these downstream molecules.

Besides c-myc, transcription factors such as E2F1, E2F2, and E2F3 induce the transcription of the miR-17-92 cluster [42, 51]. This activity might explain the discrepancy between uPAR and miR-17-5p/20 expression in BT-474 cells. Several lines of evidence indicate a functional link between the Myc and the Rb/E2F pathways, which are integrated into the control of the cell cycle. E2Fs, especially E2F3, might be important downstream effectors in Myc-induced oncogenic signaling [53-54]. Moreover, c-myc, E2F1, E2F2, and E2F3 are inhibited by the miR-17-92 cluster, and the E2F/Myc/miR-17-92 pathway forms a negative feedback loop [27, 42, 43]. Therefore, E2Fs are possibly involved in uPAR-induced miR-17-92 expression, which deserves further investigation.

We observed that uPAR RNAi in MDA MB 231 cells led to decreased protein levels of DR4 and DR5 but not decreased mRNA levels (Figure 1C and 1D), whereas miR17-5p/20a inhibited both the mRNA levels and the protein levels in transfection experiments (see Figure 2). This outcome might occur because the uPAR depletion-induced decrease (approximately 50%, see Figure 5B) of miR-17-5p and miR-20a preferentially reduces DR4/5 translation efficiency, because transfection of miR-17-5p/20a inhibitor caused more evident changes in protein levels of DR4/5 than in their mRNA levels (Figure 2D and 2E). This possibility merits further investigation.

Our data demonstrate that uPAR induces miR-17-5p/20a expression via c-myc in breast cancer and DR4 and DR5 are suppressed by miR-17-5p/20a. Our study represents an effort to address the underlying mechanism of uPAR-induced apoptosis in breast cancer. Our results underscore the potential of miR-17-5p/20a as an option for tailored therapy of breast cancers, including TNBC. Given that miR-17-5p/20a are expressed predominantly in malignant cells but not in normal cells, our study supports the notion that the inhibition of miR-17-5p/20a activity might provide a novel therapeutic approach for uPAR-overexpressing breast cancer.

## MATERIALS AND METHODS

### Reagents and antibodies

Mimics and inhibitors of miR-17-5p or miR-20a, cholesterol-conjugated miR-17-5p and miR-20a inhibitors, c-myc, uPAR, and DR5-specific small interfering RNA (siRNA) were chemically synthesized by RiboBio Co., Ltd. (Guangzhou, China). The sequence of siRNAs for c-myc, uPAR, and DR5 were as follows: c-myc, 5′-CTATGACCTCGACTACGAC-3′; uPAR, 5′-GCTGTACCCACTCAGAGAA-3′; DR4 5′-CUCUGAUGCUGUUCUUUGAtt and DR5, 5′-AAGACCCUUGUGCUCGUUGUC-3′. The following reagents and antibodies were obtained as indicated: DR5 antibody was from Santa Cruz Biotechnology (Dallas, TX,, USA); procaspase 8, caspase 3/8, and total and phosphorylated forms of ERK antibodies were from Cell Signaling Biotech; uPAR antibody was from R&D Systems (Minneapolis, MN, USA); and c-myc, actin, GAPDH, and horseradish peroxidase (HRP)-conjugated secondary antibodies were from Zhongshan Goldenbridge Biotechnology (Beijing, China). The ECL-Plus chemiluminescence system was from Applygen Technologies (Beijing, China).

### Plasmid construct

The DR4 and DR5 3′untranslated region (UTR), which contains miR-17-5p or miR-20a binding sites, was amplified by PCR. The DR4 and DR5 primers used were as follows: DR4-F, 5′-GCTCTAGACTCGAGGGATGCCTTGTATGCAA-3′ and DR4-R, 5′-GCTCTAGAGATGTTGGTCAGGCTGGT-3′; DR5-F, 5′-GCTCTAGACTCGAGGTGTGATTCTCTTCAGG-3′ and DR5-R, 5′-GCTCTAGACAGCCTGGGAGACAGAGT-3′. The PCR products were cloned into the pGL3 vector, and the recombinant plasmids (DR4-UTR-wt and DR5-UTR-wt) were mutated in the seed sequences of miR-17-5p or miR-20a by site-directed mutagenesis. These plasmids were designated DR4-UTR-mu and DR5-UTR-mu.

### Cell culture and transfection

The breast cancer cell lines MDA MB 231, MCF7, SKBR3, ZR 751, and BT 474 were obtained from the ATCC (Manassas, VA, USA). The 293T cell line was obtained from the ATCC (Manassas, VA, USA). The cell lines were cultured in 1640 medium supplemented with 10% fetal bovine serum, 25 mg/mL streptomycin, and 100 IU/mL penicillin. Cells were transfected with miRNA mimic, inhibitor, uPAR siRNA, or 2 μg plasmid by use of Lipofectamine 2000 reagent (Invitrogen, Carlsbad, CA, USA). Each treatment was performed at least three times.

### Real-time PCR

Total RNA was extracted with TRIzol Reagent (Waltham, MA, USA), mRNA levels were quantified by use of the SYBR Green Premix Reagent (Takara Bio Inc., Shiga, Japan), and microRNA levels were detected by use of a TaqMan microRNA kit (Applied Biosystems, Foster City, CA, USA) following the manufacturers’ protocols. The endogenous control glyceraldehyde-3-phosphate dehydrogenase (GAPDH) or U6 was used for normalization.

### Luciferase reporter assays

The 293T cells were co-transfected with DR4-UTR-wt (DR5-UTR-wt) or DR4-UTR-mu (DR5-UTR-mu), pRL-TK plasmid, and miR-17-5p/miR-20a or a randomized oligonucleotide as a control. At 36 hours after transfection, luciferase activities were detected by use of the Dual Luciferase Reporter Assay System (Promega, Madison, WI, USA).

### Cell growth and cell apoptosis analysis

CCK-8 experiments were performed as previously described [18]. Similarly, cell apoptosis experiments were performed in MDA MB 231 cells and MCF 7 cells by use of an Annexin V-FITC/PI Apoptosis Detection Kit (Invitrogen, Carlsbad, CA, USA) by flow cytometry. At 48 hours after transfection with miR-17-5p/20a, miR-17-5p/20a inhibitor, or a randomized oligonucleotide as a control, cells were treated with 50 ng/mL TRAIL for additional 8 hours, and cellular apoptosis was detected.

### Western blotting assay

Western blotting analysis was performed according to our previous description [20].

### Animal studies

Female BALB/c-nu mice (5 to 6 weeks old) were purchased from Vital River Laboratories (Beijing, China). The mice were subcutaneously inoculated with 7 × 10^6^ MDA MB 231 cells (0.1 mL PBS). When the tumor volume reached approximately 150 mm^3^, the mice were randomly divided into two groups (5 mice/group). The first group received an intratumor injection of antagomir-control, and the second group received an intratumor injection of antagomir-17-5p (10 nmol/mouse) and antagomir-20a (10 nmol/mouse) five times every 3 days. Tumor growth was monitored at 3-day intervals, and tumor size was calculated by the formula Tv = (L × W^2^)/2. Mean tumor weights were analyzed at day 30. Three days after the last intratumor injection, all mice were sacrificed, and the tumors were excised. The tumor tissues were stored at −80° C for RNA extraction and protein lysis. All animals received humane care, and the study of the mice was in strict accordance with the regulations of the Institute of Microbiology, Chinese Academy of Sciences of Research Ethics Committee. The protocol was approved by the Research Ethics Committee (Permit No. PZIMCAS2011001).

### Statistical analysis

Differences between groups were determined by two-tailed Student’s *t*-tests. A value of *P* < 0.05 was considered significant. Association between variables was assessed by Spearman’s nonparametric correlation.

## SUPPLEMENTARY MATERIALS FIGURES


