# Supplementary material for: Urokinase-type plasminogen activator receptor inhibits apoptosis in triple-negative breast cancer through miR-17/20a suppression of death receptors 4 and 5
Source: Oncotarget. 2017 Aug 24;8(51):88645–57. doi: 10.18632/oncotarget.20435 (PMC5687634; doi:10.18632/oncotarget.20435)
Supplement: Supplementary file 1 [file oncotarget-08-88645-s001.pdf]

Urokinase-type plasminogen activator receptor inhibits apoptosis in triple-negative breast cancer through miR-17/20a suppression of death receptors 4 and 5

SUPPLEMENTARY MATERIALS

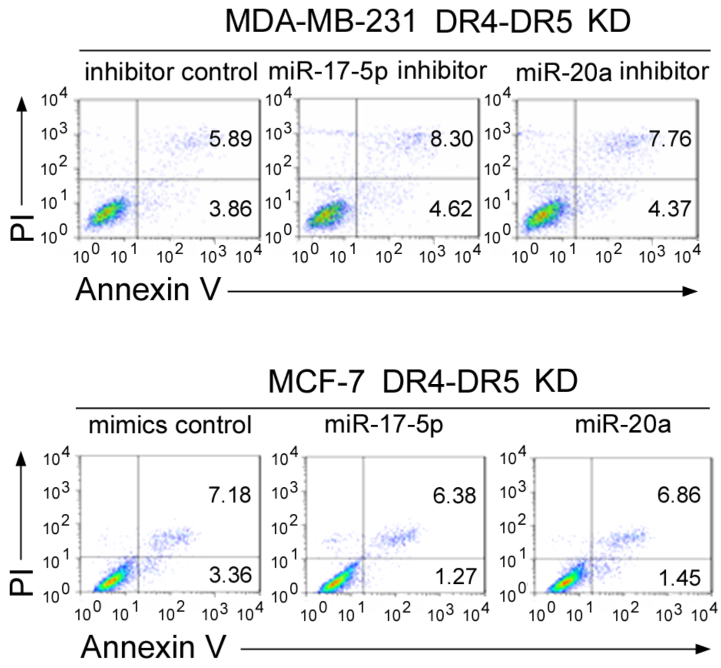

Supplementary Figure 1: MDA MB 231 cells were co-transfected with DR4/DR5 siRNA and miR-17-5p or miR-20a inhibitor or a randomized oligonucleotide as an inhibitor control, and MCF-7 cells were co-transfected with DR4/DR5 siRNA and miR-17-5p or miR-20a mimic or a randomized oligonucleotide as a mimic control. At 48 h after transfection, cells were treated with 50 ng/mL TRAIL for an additional 8 h, and then apoptotic MDA MB 231 cells (left) and MCF-7 cells (right) were detected by FACS as in Figure 1A (A).

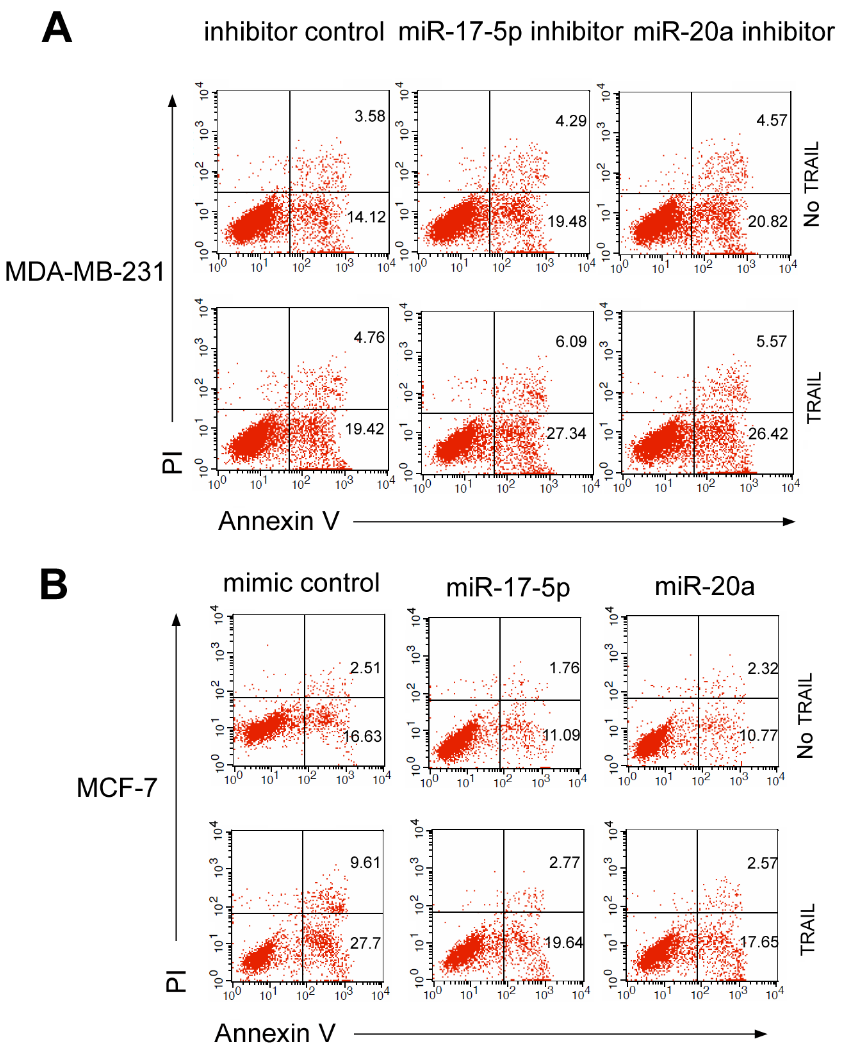

Supplementary Figure 2: MDA MB 231 cells were transfected with miR-17-5p or miR-20a inhibitor or a randomized oligonucleotide as an inhibitor control, and MCF-7 cells were transfected with miR-17-5p or miR-20a mimic or a randomized oligonucleotide as a mimic control. At 48 h after transfection, cells were treated with or without 50 ng/mL TRAIL for an additional 48 h, and apoptotic MDA MB 231 cells **(A)** and MCF-7 cells **(B)** were detected by FACS as in Figure 1A.
